# Peroneal nerve palsy caused by a synovial cyst of the proximal tibiofibular joint: a report of two cases and review of the literature

**DOI:** 10.11604/pamj.2019.34.115.18339

**Published:** 2019-10-29

**Authors:** Fekih Aymen, Saadana Jacem, Othman Youssef, Aloui Issam, Abid Abderrazek

**Affiliations:** 1 Department of Trauma and Orthopaedics Surgery, Fattouma Bourguiba University Hospital, Monastir, Tunisia

**Keywords:** Synovial cyst, ganglion cyst, proximal tibiofibular joint, peroneal nerve, treatment

## Abstract

Synovial cyst of the proximal tibiofibular joint is a very rare condition, for which there is no consensus regarding treatment. Two macroscopic forms may be encountered: extraneural cysts and intraneural cysts. We present the cases of two patients who had synovial cysts of proximal tibiofibular joint that caused peroneal nerve palsy. We discussed the special features of synovial cysts and reviewed the literature. We considered the best treatment of synovial cysts originating from proximal tibiofibular joint and causing peroneal nerve palsy to be a total surgical removal as soon as possible after the diagnosis is made. However, follow-up is needed because recurrence is possible. It should be kept in mind that despite surgical treatment the neurological symptoms may not recover.

## Introduction

Synovial cysts are benign soft-tissue tumors that arise from synovial joints or tendon sheaths. They are usually seen in popliteal and dorsal wrist localizations. The Proximal Tibiofibular Joint (PTFJ) is an unusual localization for synovial cysts, first reported by Lenander in 1891 [1]. Synovial cysts of PTFJ can be symptomatic by the compression they exert on the adjacent structures including the peroneal nerve which is a rare conflict [2, 3]. Two forms of this compression are described: a first, infiltrating and fusing with the nervous tissue, this is the intraneural cyst, and another exerting a compressive action on the nerve without infiltrating it, it is the extraneural cyst [3]. We report two cases of synovial cysts originating from the PTFJ which caused peroneal nerve palsy.

## Patient and observation

**Figure 1:**
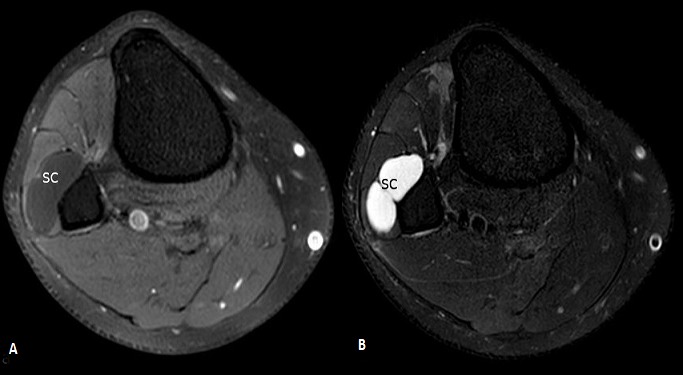
A) axial view of preoperative MRI showing the synovial cyst (SC) hypointense on T1; B) hyperintense on T2-weighted sequence

**Figure 2:**
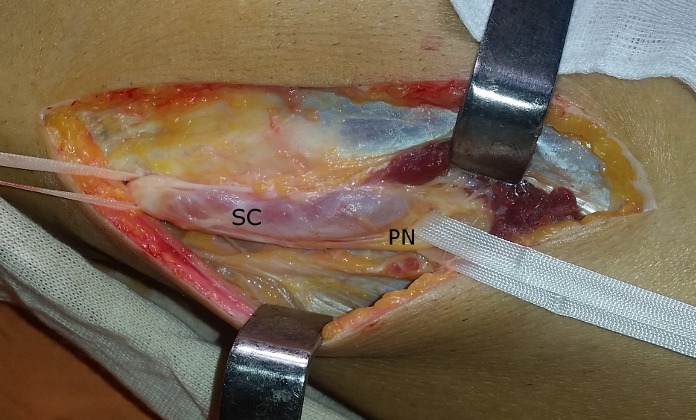
Intraoperative appearance of the synovial cyst (SC) that infiltrates and compresses the peroneal nerve (PN)

**Figure 3:**
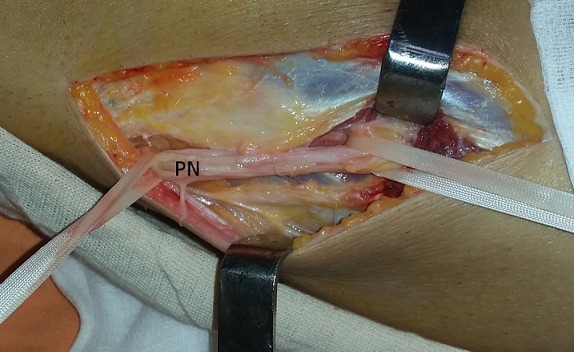
Intraoperative view of the peroneal nerve (PN) after cyst resection

**Figure 4:**
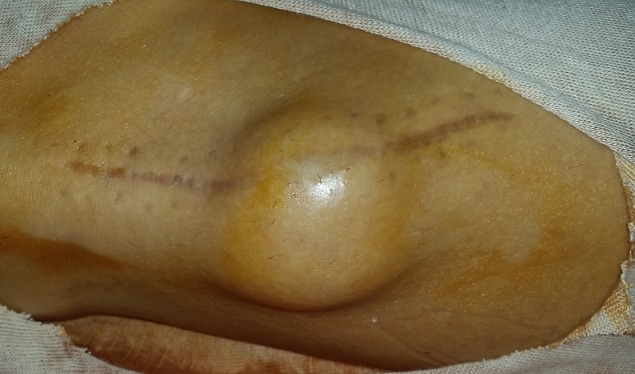
Recurrence of the synovial cyst four months after the first surgery

**Figure 5:**
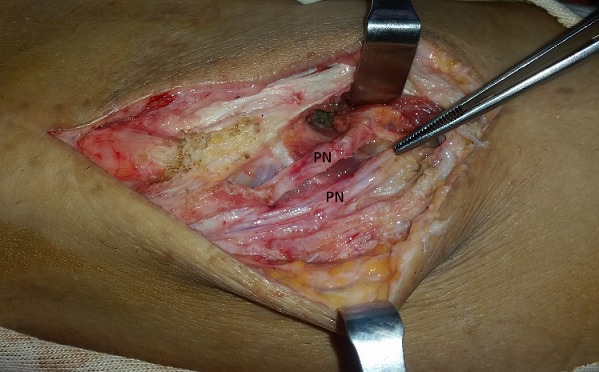
Intraoperative view showing total resection of the cyst and the dissociation of the peroneal nerve (PN) fibers

A 42-year-old man consulted the neurosurgery department complaining of slight back pain and right drop-foot. His physical examination showed paresis of the tibialis anterior, lateral peroneal and extensor digitorum muscles with a muscle strength grade 2. Diagnosis was thought as a lumbar disc herniation and Magnetic Resonance Imaging (MRI) was carried out. It showed bulging of L4-L5 and L5-S1 and no disc extrusion. Electromyographic investigation showed that the common peroneal nerve was affected at the knee level. The patient was referred to our orthopedic department. He had neither knee pain nor history of trauma to his knee. Physical examination of right knee showed no abnormality other than tenderness around the fibular head. He had no palpable mass. The radiography was normal. MRI of the knee revealed a 48×20×12mm soft tissue mass with clear borders localized at the anterior of proximal tibiofibular joint with poor visualization of the common peroneal nerve (Figure 1). Intraoperatively, the cystic mass had infiltrated the common fibular nerve with dissociation of its fibers and without any real visualization of pedicle with the proximal tibiofibular joint (Figure 2). Resection was minitious to avoid nerve injury (Figure 3). Histopathological investigation had revealed as a synovial cyst. Unfortunately the cyst recidivated four months later with reappearance of a larger mass of about 5cm at the same localization (Figure 4). Surgical resection removed the entire cyst and the pedicle was found and cut, the nerve appears continuous but its fibers are dissociated (Figure 5). Neurological status of the patient at 18 months after the second operation showed a slight improvement despite continued rehabilitation. We found no recurrence of the synovial cyst on MRI at the last 18-month follow-up.

**Figure 6:**
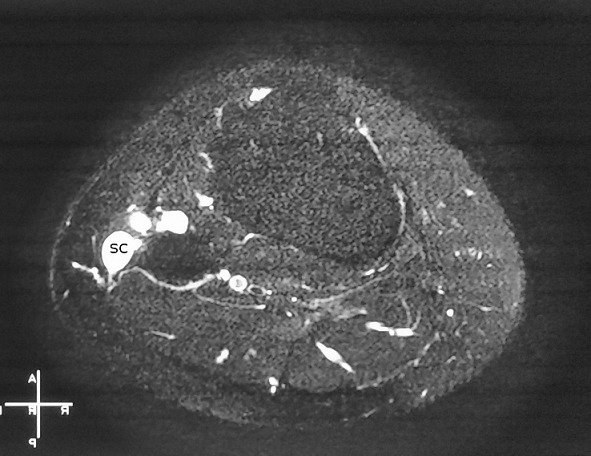
Axial view of preoperative MRI showing the multicompartmental synovial cyst (SC)

**Figure 7:**
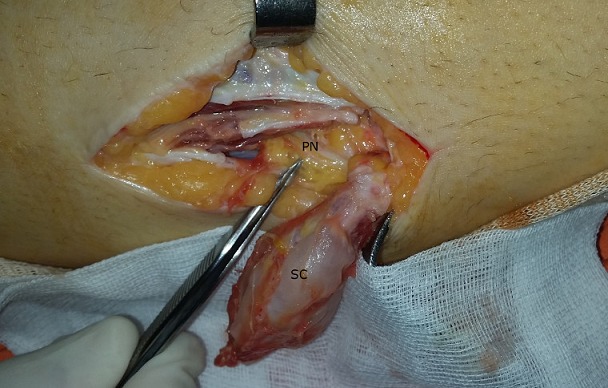
Intraoperative view showing the close contact between the synovial cyst (SC) and the peroneal nerve (PN)

The second case is about a 29-year-old woman who was admitted to the orthopedic department complaining of swelling and pain at her left knee and total loss of ankle active dorsiflexion. She had symptoms for 2 months and no history of trauma. Physical examination of the knee showed slight swelling at the fibular head but no palpable mass. In neurological examination, all motor functions of common peroneal nerve were lost. MRI showed an 85×24×10mm multi-compartmental cystic formation at the anterolateral side of fibular head and communicating at its superior pole with the proximal tibiofibular joint by a thin 2mm pedicle (Figure 6). Intraoperatively, cystic mass that adhered to and compressed the common peroneal nerve (Figure 7) was totally excised and the pedicle was found and cut. Anatomopathology confirmed the synovial type of the cyst. Peroneal nerve palsy was completely improved 12 months after operation. No recurrence was seen at 2 years of follow-up.

## Discussion

According to the recent literature, only 73 cases of peroneal nerve palsy caused by cysts of the Proximal Tibiofibular Joint (PTFJ) have been reported since 1981 [4]. However, differences in histological definitions and artificial distinctions that exist between synovial cysts and ganglion have complicated comparison of published data [5]. Synovial cysts of the PTFJ are rare [6, 7]. Ilahi *et al.* in a study of 645 MRIs knees, found that their prevalence was 0.76% [6]. Pediatric cases are rare and there are seven of which only three are extraneural cysts [3]. The pathophysiology of these cysts is poorly understood with several opposed theories to differentiate between intraneural and extraneural cysts. It seems to be a single entity at two different times of its natural history [3]. Initially, the cyst is extraneural. Compression of the nerve by the cyst would result in adhesion, then a fusion with the epineurium and an intraneural penetration of the tumor. Subsequently, one would observe a degeneration of nerve fascicles [8]. Synovial cysts may be primary or secondary to degenerative or traumatic pathology of the corresponding joint [9]. Schwimmer *et al.* [10] stated that increased synovial fluid production secondary to trauma or arthritis may cause distension of synovial bursa or herniation of distended capsule, and that this process may be responsible for the formation of synovial cysts. In contrast of this theory, a pedicle was found between the cyst and the joint only in 20-50% of the patients [11, 12]. We found this pedicle connection between the cyst and the joint in our two cases. Synovial cysts can produce neuropathy either gradually or suddenly by direct pressure on the nerve. The clinical presentation of synovial cysts is variable. When the cysts grows slowly in size, the condition can remain undetected until the patient presents with calf atrophy and weakness of the affected muscles [13]. On examination, a slowly growing swelling and pain are the main symptoms [2]. If the mass compresses the nerve as in our patients, a neurological deficit which is motor rather than sensory is observed [14, 15]. If the patient has no knee pain, and drop-foot is the only finding, as in our first case, suggesting S1 root compression, this clinical presentation may mislead the physician.

The imaging method for diagnosis and differential diagnosis is MRI [16, 17]. The cystic lesion appears hypo or iso intense, with diffuse or peripheral enhancement of contrast after the gadolinium injection [3]. Magnetic Resonance Imaging (MRI) provides a better assessment of regional anatomical relationships, including the communication to the joint through a pedicle and the extra or intraneural location of the cyst [4, 18, 19]. Ultrasonography is another imaging method for soft tissue masses and may be helpful in unclear cases [17] but this one has some limitations such as difficulty in benign-malignant differentiation and detecting the communication between the cyst and the joint. Ultrasonography may be helpful for follow-up after treatment [20]. Synovial cysts can be difficult to differentiate from malignant nerve tumors, particularly peripheral nerve tumors like neurosarcoma or malignant schwannoma [13]. Synovial cysts can be treated conservatively by aspiration of the cyst and steroid injection if the patient does not have neurological symptoms, but the recurrence rate of this therapy regimen is very high [14]. Total surgical removal of the cyst is the most appropriate treatment method in patients who have neurological symptoms or recurrent cysts after conservative therapy [2]. In the case of intraneural cyst, the accurate dissection made possible by the operating microscope often permits total removal of the cyst without damage to the nerve fascicles, thus providing the conditions for good functional recovery [21]. This recovery is reported in the literature within 45 days to 1 year after complete and non-mutilating excision of the nerve [22]. Despite surgical excision, neurological symptoms sometimes do not recover. We obtained total recovery in case 2 but neurological symptoms (drop-foot) in case 1 were still persistent without recurrence of the cyst after the second surgery. The recurrence rate after surgical resection is less than 10% [12, 22, 23].

## Conclusion

Peroneal nerve palsy caused by a synovial cyst of the Proximal Tibiofibular Joint (PTFJ) is a very rare disease which must be considered if the patient presents neurological abnormalities of the lower leg. Although there is no consensus on the treatment of this rare condition, we think that total surgical removal of the cyst is the best alternative if the patient has neurological symptoms. The pedicle should be cut to save the patient from recurrence. Recovery rates are sometimes unclear since they depend on the date of the operative treatment and on the kind of preexisting nerve injury.

## Competing interests

The authors declare no competing interests.
